# Enterovirus D68 in Hospitalized Children, Barcelona, Spain, 2014–2021

**DOI:** 10.3201/eid2807.220264

**Published:** 2022-07

**Authors:** Cristina Andrés, Jorgina Vila, Anna Creus-Costa, Maria Piñana, Alejandra González-Sánchez, Juliana Esperalba, Maria Gema Codina, Carla Castillo, Maria Carmen Martín, Francisco Fuentes, Susana Rubio, Karen García-Comuñas, Rodrigo Vásquez-Mercado, Narcís Saubi, Carlos Rodrigo, Tomàs Pumarola, Andrés Antón

**Affiliations:** Hospital Universitari Vall d’Hebron, Vall d’Hebron Research Institute, Barcelona, Spain (C. Andrés, J. Vila, A. Creus-Costa, M. Piñana, A. González-Sánchez, J. Esperalba, M.G. Codina, C. Castillo, M. Carmen Martín, F. Fuentes, S. Rubio, K. García-Comuñas, R. Vásquez-Mercado, N. Saubi, C. Rodrigo, T. Pumarola, A. Antón);; CIBER de Enfermedades Infecciosas, Instituto de SaludCarlos III, Madrid, Spain (C. Andrés, M. Piñana, A. González-Sánchez, J. Esperalba, M.G. Codina, N. Saubi, T. Pumarola, A. Antón);; Hospital Universitari Germans Trias i Pujol, Barcelona (C. Rodrigo)

**Keywords:** Enterovirus D68, EV-D68, genetic diversity, surveillance, pediatric disease, viruses, Barcelona, Spain, respiratory infections

## Abstract

To determine molecular epidemiology and clinical features of enterovirus D68 (EV-D68) infections, we reviewed EV-D68–associated respiratory cases at a hospital in Barcelona, Spain, during 2014–2021. Respiratory samples were collected from hospitalized patients or outpatients with symptoms of acute respiratory tract infection or suggestive of enterovirus infection. Enterovirus detection was performed by real-time multiplex reverse transcription PCR and characterization by phylogenetic analysis of the partial viral protein 1 coding region sequences. From 184 patients with EV-D68 infection, circulating subclades were B3 (80%), D1 (17%), B2 (1%), and A (<1%); clade proportions shifted over time. EV-D68 was detected mostly in children (86%) and biennially (2016, 2018, 2021). In patients <16 years of age, the most common sign/symptom was lower respiratory tract infection, for which 11.8% required pediatric intensive care unit admission and 2.3% required invasive mechanical ventilation; neurologic complications developed in 1. The potential neurotropism indicates that enterovirus surveillance should be mandatory.

In 1962, enterovirus D68 (EV-D68) was first isolated from the oropharynx of children in California, USA, who were hospitalized for lower respiratory tract infection (LRTI) (*1*). Although infections can occur at any age, children are the most susceptible to enterovirus infections (*2*). In temperate countries, enterovirus circulation usually follows a seasonal pattern, peaking in late summer and early autumn, but a second peak can also be detected during spring (*3*).

Until 2007, EV-D68 was rarely implicated in severe diseases and was poorly detected, associated only with small outbreaks in the United States and the Netherlands (*4*,*5*). However, in 2014, EV-D68 gained attention because of a large outbreak in the United States that was associated with severe respiratory illness and, in some cases, with neurologic complications, such as acute flaccid paralysis (AFP) (*6*). In Europe, circulation of EV-D68 was low and mild, but circulation increased in the following years, especially in 2021, after preventive measures for SARS-CoV-2 were eased (*7*). We reviewed EV-D68–associated respiratory cases, particularly in children, diagnosed at a tertiary-care university hospital in Barcelona (Catalonia, Spain) during 2014–2021. Institutional review board approval (PR(AG)173/2017) was obtained from the HUVH Clinical Research Ethics Committee.

## Materials and Methods

### Patients and Samples

During October 2014–November 2021, upper and lower respiratory tract specimens were collected by hospital staff and sent to the Respiratory Viruses Unit of the Vall d’Hebron University Hospital laboratory for confirmation of respiratory viruses. Samples were taken according to clinical criteria from patients with suspected acute respiratory tract infection or enterovirus infection who were hospitalized or sought care at the emergency department. In addition, since September 2021, respiratory samples for SARS-CoV-2 screening were further tested for other respiratory viruses. We retrospectively collected patient demographic features (sex and age) for all laboratory-confirmed cases of enterovirus infection and collected clinical data only for patients <16 years of age (pediatric population).

Regarding the clinical definitions used, upper respiratory tract infections (URTIs) were infections from the nose to the larynx; LRTIs were recurrent wheezing, asthma, bronchiolitis, and pneumonia. To ensure that the length of hospital stay or respiratory support were associated only with EV-D68, we studied LRTI severity in patients requiring admission because of respiratory tract infection. Respiratory support was divided into 5 groups: none, oxygen through nasal cannula, high-flow nasal cannula, noninvasive mechanical ventilation, and invasive mechanical ventilation. EV-D68–associated AFP was defined as myelitis causing sudden onset of paralysis with T2 hyperintensity in medulla gray matter with dorsal brain stem variably affected on magnetic resonance images and EV-D68 detected in respiratory specimens.

### Enterovirus Detection and Characterization

We performed enterovirus detection by using specific real-time multiplex reverse transcription, as previously described (*8*). The characterization of enterovirus was performed by the phylogenetic analyses of the partial viral protein 1 (VP1) coding-region according to the protocol recommended by the World Health Organization, with minor modifications (*8*).

### Statistical Analyses 

We performed statistical analysis by using SPSS version 22 (SPSS Inc., https://www.ibm.com). To assess associations between categorical variables, we performed χ^2^ testing and calculated Z scores. We considered p<0.05 to be significant.

## Results

Over the 7 years of the study, 67,798 respiratory specimens (39,183 patients) were received for laboratory confirmation of respiratory viruses. A total of 1,423 (2%) samples from 1,313 (3%) patients were laboratory confirmed as containing enterovirus. Phylogenetic analysis of the partial VP1 coding region revealed that 187 (13%) of the 1,423 strains from 184 (14%) of the 1,313 patients were EV-D68 (147 subclade B3, 80%; 32 newly emerged subclade D1, 17%; 2 subclade B2, 1%; and 1 subclade A, <1%) (Appendix Figure 2). EV-D68 was detected mostly in pediatric populations (158/184; 86%) (median age 3 years; interquartile range 1.73–6 years; age range 8 months to 77 years), especially in patients <5 years of age (117/158; 74%). The distribution of EV-D68 infections (Appendix Figure 1) was like that of other enteroviruses; circulation peaked in autumn and spring, especially during 2016, 2018, and 2021; fewer cases were reported in 2015, 2017, 2019; and no cases were reported in 2020. Circulation of strains belonging to the several subclades shifted throughout the study period; B3 predominated until 2017, and B3 and D1 co-circulated until 2021, when B3 was predominant (Table 1; Appendix Figure 1). Moreover, the distribution of these clades among the studied population differed (p<0.00001) (Appendix Table). B3 was detected mostly among the pediatric population (<16 years of age, 95% of cases), whereas subclade D1 was detected equally in pediatric and adult (>16 years) populations (17/32 [53%] vs. 15/32 [47%]; p<0.00001).

**Table 1:** Distribution of enterovirus D68 subclades, by year, for all patients and hospitalized children, Barcelona, Spain, 2014–2021*

Clade	Year								
	2014	2015	2016	2017	2018	2019	2020	2021	Total
All									
A	1								1
B2		1	1						2
B3		6	26		24			91	147
D1	4			3	20	2		3	32
Hospitalized children									
A									
B2		1	1						2
B3		6	21		17			20	64
D1	3			1	5				9
Total	5	7	27	3	44	2		94	

*Blank cells indicate zero.

Among the 158 children with EV-D68, 76 (48%) were hospitalized and 82 (52%) were seen as outpatients (Table 2). Until 2021, a total of 12/82 (15%) patients were outpatients, compared with 70/82 (85%) during 2021.

**Table 2:** Demographic and clinical characteristics of patients in study of enterovirus D68 in hospitalized children, Barcelona, Spain, 2014–2021*

Characteristic	Hospitalized, no. (%)†	Outpatient, no. (%)
Sex		
M	44 (57.9)	47/82 (57.3)
F	56 (42.1)	35 (42.7)
Age, y		
<2	24 (31.6)	24 (29.3)
2–4	34 (44.7)	34 (41.4)
>5	18 (23.7)	24 (29.3)
Signs/symptoms‡		
LRTI	56 (73.6)	45 (54.9)
>24 mo	40 (71.4)	36 (80.0)
<24 mo	16 (28.6)	9 (20.0)
URTI	10 (13.2)	27 (32.9)
Other	10 (13.2)	10 (12.2)
Treatment for LRTI		
Chronic respiratory comorbidities	28/56 (50)	20/45 (44.4)
Asthma-directed therapies		
β2 agonists	52/56 (92.9)	43/45 (95.6)
Systemic corticosteroids	51/56 (91.1)	34/45 (75.6)
Hospitalization for LRTI		
Hospital length of stay, d§	3 (1–5)	NA
Respiratory support§	44 (78.6)	NA
Maximum respiratory support required¶		
Conventional oxygen	23 (52.3)	NA
HFNC	13 (29.5)	NA
NIMV	6 (13.6)	NA
IMV	1 (2.3)	NA
ECMO	1 (2.3)	NA
Duration of respiratory support§¶#	3 (1–4)	
PICU admission	9 (11.8)	NA
PICU length of stay, d§	4 (2–9)	NA

*Units of measure are no. (%) unless otherwise indicated. ECMO, extracorporeal membrane oxygenation; HFNC, high-flow nasal cannula; IMV, invasive mechanical ventilation; LRTI, lower respiratory tract infection; NA, not applicable; NIMV, noninvasive mechanical ventilation; PICU, pediatric intensive care unit; URTI, upper respiratory tract infection. †Percentages are calculated vertically, according to the total cases. ‡The main symptom at time of hospital admission or consultation. §For continuous variables, means and interquartile ranges are indicated. ¶Three patients received home mechanical ventilation and required increased respiratory support during hospitalization. #Excludes the 3 patients with home mechanical ventilation and the patient who received ECMO.

With regard to clinical signs and symptoms, most common were LRTI (101/158; 64%), followed by URTI (37/158; 23%). A total of 9/158 (6%) pediatric patients had fever as the only sign, 6/158 (4%) had gastroenteritis, and 1 (1%) had myelitis and AFP (a 2-year-old girl with no underlying diseases but not fully recovered with quadriplegia and respiratory failure requiring home tracheotomy mechanical ventilation and feeding through gastrostomy). The remaining (4/158; 2.5%) patients were asymptomatic. 

## Discussion

Interest in EV-D68 was limited until the large outbreak that occurred in the United States in 2014 (*6*,*9*). Although EV-D68 circulation had been previously described, that large outbreak affecting mainly children was associated not only with severe respiratory disease but also with neurologic complications in some cases. Furthermore, the circulating EV-D68 strains belonged to previously circulating lineages, and therefore, there was no clear evidence of a new virus strain associated with increased severity (*6*,*9*). Nevertheless, during the same period, further studies began not only in the United States but also in Europe to monitor EV-D68 circulation (*10*). Results revealed a low level of EV-D68 detection and milder clinical manifestations in Europe compared with those in the United States (*10*). Similarly, the EV-D68 circulation in Barcelona was low during that period (*8*). However, in the following seasons, the trend increased, particularly during 2016 and 2018, as reported in other regions of Spain (*11*) and Europe (*12*,*13*), especially the upsurge observed during the 2021–22 season (*7*). According to those data, EV-D68 seems to follow a biennial circulation pattern as recently defined (*14*,*15*), which was displaced during 2020 because of the COVID-19 pandemic, but higher numbers of cases were detected during 2021 (*7*).

Four distinct clades of EV-D68 (A–D) have been described (*16*) in addition to subclades A, B1, B2, and B3 (*10*,*17*). Clades cocirculated variably; B3 predominated during the studied seasons, which is in concordance with other reports (*10*,*18*). Moreover, in our study, viruses belonging to the new emerging subclade D1 within clade D, were mainly detected during 2018, and cocirculation with subclade B3 was equal until 2018, as recently reported around Europe (France and Italy) (*12*,*19*). Furthermore, subclade D1 was observed similarly among pediatric and adult populations, compared with B3, which was mostly detected in children. This age effect depending on clade, in concordance with our results, has been reported in other studies (*19*,*20*). Of note, a recent study reported changes in the VP1 region of D1 associated with lower cross-protection in adults (*21*).

The most common clinical features of EV-D68 infection in children in this study were respiratory, and AFP developed in only 1 patient. EV-D68 has been mostly associated with LRTI in children >2 years of age, 50%–70% of whom previously had asthma or recurrent wheezing and 20% of whom had no comorbidities, as described in our study. In addition, most cases from our study were mild; few patients were admitted to intensive care units (ICUs). However, during the US outbreak in 2014, respiratory signs/symptoms reported for hospitalized patients were more severe: 59% of patients required ICU admission, 23% noninvasive mechanical ventilation, and 8% invasive mechanical ventilation; therefore, previous asthma or reactive airway disease might increase the risk for ICU admission and the need for ventilatory support (*22*). Despite changes in the virologic properties of circulating viruses, the clinical features remained similar to those reported in 2014 in Europe, in contrast to the United States and Canada. In addition, during the US outbreak, AFP cases associated with EV-D68 infection (subclade B3), in which this virus was the only pathogen isolated, increased (*6*,*9*). Although enteroviruses are known to be neurotropic, we detected only 1 case of AFP associated with EV-D68 subclade B3 infection. 

The potential neurotropism of EV-D68 and other enteroviruses suggests that surveillance should be mandatory, which is one of the aims of the European Non-Polio Enterovirus Network (https://www.escv.eu/enpen). The year-round circulation of EV-D68 should help with close monitoring of this enterovirus, as well as prompt response to the potential occurrence of outbreaks and related clinical burden.

Appendix Additional information for study of enterovirus D86 in hospitalized children, Barcelona, Spain, 2014–2021.
