# Supplementary material for: Enterovirus D68 in Hospitalized Children, Barcelona, Spain, 2014–2021
Source: Emerg Infect Dis. 2022 Jul;28(7):1327–31. doi: 10.3201/eid2807.220264 (PMC9239859; doi:10.3201/eid2807.220264)
Supplement: Appendix — Additional information for study of enterovirus D86 in hospitalized children, Barcelona, Spain, 2014–2021. [file 22-0264-Techapp-s1.pdf]

Article DOI: https://doi.org/10.3201/eid2807.220264

Enterovirus-D68 in Hospitalized Children, Barcelona, Spain, 2014–2021

Appendix

Appendix Table. Age distribution of the different EV-D68 subclades B3 and D1 among all cases and hospitalized children

Age groups	Tested cases	EV laboratory-confirmed cases	%*	EV-D68	%†	Subclades (all)				Paediatric EV-D68		Subclades (hospitalized)			
						B3	%‡	D1	%‡	No.	%‡	B3	%§	D1	%§
<2 y	8259	419	5,1%	50	11,9%	41	28	8	25	49	98,0%	19	39%	5	10%
2–4 y	4887	640	13,1%	68	10,6%	63	43	3	9	66	97,1%	29	44%	1	2%
5–14 y	5248	175	3,3%	41	23,4%	35	24	6	19	41	100,0%	15	37%	3	7%
15–64 y	10709	66	0,6%	19	28,8%	7	5	10	31	2	10,5%	1	50%	-	-
>64 y	10080	13	0,1%	6	46,2%	1	<1	5	16	-	-	-	-	-	-
TOTAL	39183	1313	3% p<*0.00001*	184	14%	147	80%	32	17%	158	86%	64	41%	9	6%

*Percentages are calculated horizontally according to the tested samples.
†to EV laboratory-confirmed cases.
‡to EV-D68.
§Paediatric EV-D68.

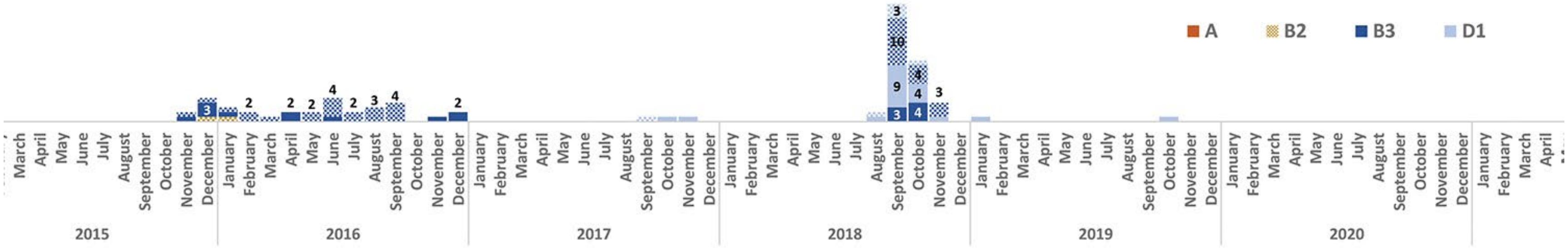

Appendix Figure 1. Monthly distribution (per year) of EV-D68 (sub)clades throughout the study period. Cases from hospitalized children are labeled as a square pattern with the same (sub)clade color. Only numbers >1 are represented.

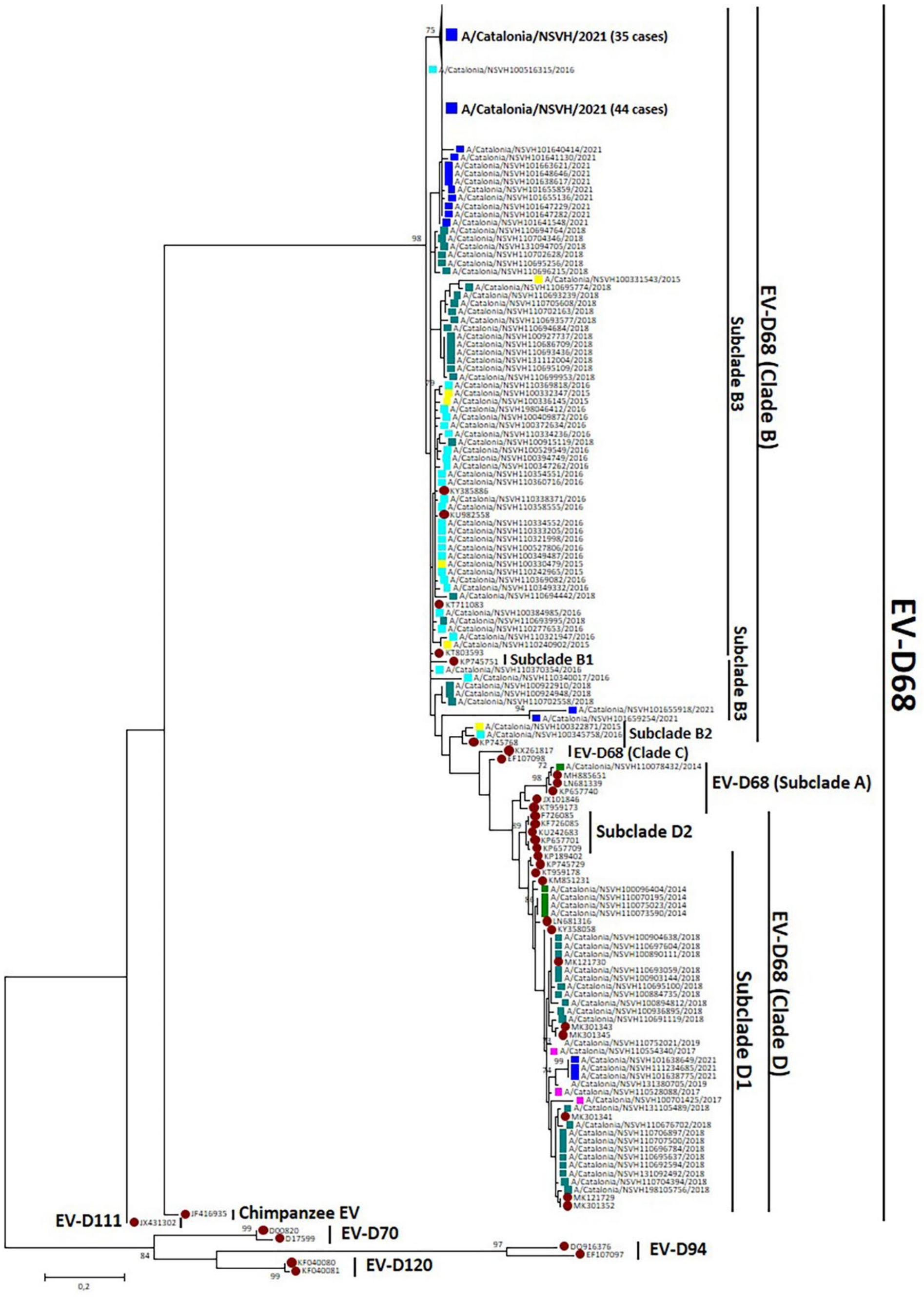

Appendix Figure 2. Phylogenetic tree of partial EV-D68 VP1 coding-region sequences. The sequences from the present study are square labeled in green (2014), in yellow (2015), in light blue (2016), in pink (2017), in turquoise (2018), in orange (2019) and in blue (2021). The reference sequences from the different clades and subclades are dot labeled in red, together with the newly emerged D1 sequences.
